# Cerebral Neurovascular Networks May Serve as Potential Targets for Identifying Disorders of Consciousness: A Synchronous Electroencephalography and Functional Near‐Infrared Spectroscopy Study

**DOI:** 10.1002/mco2.70530

**Published:** 2025-12-10

**Authors:** Nan Wang, Juanning Si, Yifang He, Jiuxiang Song, Xiaoke Chai, Dongsheng Liu, Jingqi Li, Tan Zhang, Tianqing Cao, Qiheng He, Sipeng Zhu, Yitong Jia, Wenbin Ma, Yi Yang, Jizong Zhao

**Affiliations:** ^1^ Department of Neurosurgery Peking Union Medical College Hospital, Chinese Academy of Medical Sciences and Peking Union Medical College Beijing China; ^2^ Department of Neurosurgery Beijing Tiantan Hospital, Capital Medical University Beijing China; ^3^ School of Instrumentation Science and Opto‐Electronics Engineering Beijing Information Science and Technology University Beijing China; ^4^ School of Advanced Manufacturing Nanchang University Nanchang Jiangxi China; ^5^ China National Clinical Research Center for Neurological Diseases Beijing China; ^6^ Clinical College of Neurology Neurosurgery and Neurorehabilitation Tianjin Medical University Tianjin China; ^7^ Department of Neurosurgery Tianjin Huanhu Hospital Tianjin China; ^8^ Department of Neurosurgery Aviation General Hospital Beijing China; ^9^ Hangzhou Mingzhou Brain Rehabilitation Hospital Hangzhou China; ^10^ Department of Neurosurgery The Second Affiliated Hospital of Soochow University Suzhou China; ^11^ Brain Computer Interface Transitional Research Center Beijing Tiantan Hospital, Capital Medical University Beijing China

**Keywords:** disorders of consciousness, electroencephalography, functional near‐infrared spectroscopy, neurovascular coupling, noninvasive brain–computer interfaces, resting state

## Abstract

The diagnosis and management of disorders of consciousness (DoC) remain a critical challenge in clinical medicine and neuroscience. The key bottleneck is the lack of reliable biomarkers and an incomplete understanding of the pathophysiological mechanisms that underlie DoC. In view of this, a bedside‐compatible, multimodal technique based on electroencephalography (EEG) and functional near‐infrared spectroscopy (fNIRS) was utilized to simultaneously capture neuronal oscillations and accompanying hemodynamics, so as to explore neurovascular biomarkers that can effectively discriminate different states of DoC. Resting‐state EEG‐fNIRS data from 13 regions of interest (ROIs) were acquired and compared across healthy controls (HC), minimally conscious state (MCS), and unresponsive wakefulness syndrome (UWS) groups. Hemodynamics‐based functional connectivity and the spectral power of neuronal activity were quantified and subsequently employed to interrogate neurovascular coupling. The results demonstrated significantly stronger neurovascular coupling and beta‐band power in premotor and Broca's areas of the MCS group. A multimodal classifier achieved an accuracy of 87.9% in distinguishing between MCS and UWS. The noninvasive, bedside‐suitable nature of this tool underscores its potential for routine monitoring and prognostic assessment in DoC, addressing a critical need for accessible and reliable biomarkers in both neurology and intensive‐care practice.

## Graphical abstract

Simultaneous EEG‐fNIRS recorded resting‐state activity in DoC patients. Neurovascular coupling analysis revealed elevated EEG_beta and fNIRS_HbO connectivity in PMC and Broca's regions, distinguishing MCS from UWS with high accuracy (AUC = 0.879). EEG‐fNIRS multimodal integration supports bedside monitoring.

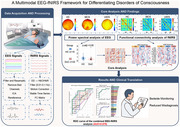

## 1 Introduction

Objective and accurate assessment of residual awareness remains one of the most critical challenges for patients with disorders of consciousness (DoC) [1, 2]. The DoC mainly includes coma (absence of both wakefulness and awareness), unresponsive wakefulness syndrome (UWS; preserved wakefulness but without awareness of themselves and the external environment), and minimally conscious state (MCS; present minimal, inconsistent, but reproducible signs of awareness) [1]. The divergent clinical trajectories of MCS and UWS, particularly the markedly higher probability of recovery in MCS, highlight the urgent need to identify objective biomarkers that can accurately quantify residual awareness in DoC. At present, the Coma Recovery Scale–Revised (CRS‐R) is regarded as the gold standard for diagnosing DoC in clinical practice. However, the clinical behavioral evaluation relies on overt behavioral responses, rendering the result subjective and error‐prone, yielding a misdiagnosis rate of approximately 41% [3, 4]. In fact, discriminating MCS from UWS is inherently difficult due to the unique characteristics of DoC: patients commonly exhibit concurrent cognitive and sensory deficits, and their level of consciousness is weak and often fluctuates markedly, collectively rendering the objective evaluation of residual awareness exceptionally challenging.

Over the past few decades, advancements in neuroimaging techniques, coupled with the growing need for clinical applications, have significantly facilitated the investigation of DoC's functional brain activity. Electroencephalography (EEG) provides direct, millisecond‐resolution measurement of neuronal electrical activity across canonical frequency bands [5]. It has been reported that patients with DoC exhibited disrupted EEG power spectra, particularly attenuated alpha (8–12 Hz) rhythms alongside enhanced delta (1–4 Hz) activity [6], which correlate strongly with the levels of consciousness and show significant differences between healthy control (HC), MCS, and UWS [7, 8, 9]. Owing to its excellent temporal resolution and portability, EEG has been utilized to detect residual consciousness, to evaluate the therapeutic effects, and to explore the underlying mechanisms of consciousness in field of DoC Task‐based experimental paradigms mainly including language tasks [10], motor imagery (MI) tasks [11], and name‐calling (subject's own name, [SON]) task [12], are employed to aid clinical diagnosis and prognosis. High‐density resting‐state EEG enables the construction of quantified brain networks, complementing systematic behavioral assessments and contributing to reduced misdiagnosis rates frequently reported in these patients [13]. Additionally, EEG is routinely used to evaluate the therapeutic efficacy of interventions such as transcranial direct current stimulation (tDCS) [14], repetitive transcranial magnetic stimulation (rTMS) [15], and spinal cord stimulation (SCS) [16] in patients with DoC [17]. However, the widespread practical value of EEG in ambulatory settings remains constrained: (1) volume‐conduction effect degrades the spatial resolution; (2) the signal is vulnerable to both electromagnetic interference and motion‐related artifacts, particularly in this population with involuntary movements [18].

Functional magnetic resonance imaging (fMRI) is a hemodynamics‐based neuroimaging technique, which indirectly reflects neuronal activity by measuring the fluctuations of blood–oxygen‐level‐dependent (BOLD) signals [19]. In patients with DoC, converging evidence has revealed disruption of the thalamo‐cortical and cortico‐cortical association fibers, hypometabolism ranging from focal to whole‐brain levels, attenuated large‐scale brain‐network connectivity, reduced interregional information exchange, and diminished efficiency and complexity of the brain [20, 21, 22]. Notably, task‐based fMRI and EEG can identify covert consciousness (i.e., cognitive‐motor dissociation, CMD) in patients who appear unresponsive on the clinical behavioral examination [23]. However, the application of fMRI in routine bedside assessment is severely hampered by its cumbersome hardware, extreme vulnerability to motion artifacts, and incompatibility with critically ill patients or those with metallic implants [24]. Alternatively, functional near‐infrared spectroscopy (fNIRS) is another indirect hemodynamics‐based neuroimaging technique that can be used for evaluating the brain functional activity by measuring concentration changes of oxygenated‐(HbO), deoxygenated‐(HbR), and total‐(HbT) hemoglobin simultaneously with higher ecological validity. fNIRS is more tolerant of movement artifacts and metal implants than fMRI, is superior to EEG in localizing and segmenting brain regions, and is not subject to interference from electrical stimulation. Owing to its unique strengths, fNIRS holds significant clinical value for diagnosing residual awareness in patients with DoC [11], particularly due to its sensitivity to disruptions within the default mode network (DMN), core neural correlate of conscious awareness in DoC. fNIRS offers valuable diagnostic value for detecting residual consciousness of patients with DoC under both resting‐state [25] and task [26] paradigms. Its utility stems particularly from sensitivity to DMN disruption, a core neural correlate of consciousness impairment in DoC [25, 27]. Furthermore, fNIRS enables identification of CMD in DoC patients through motor command execution [28]. Clinically, it evaluates therapeutic efficacy and prognosis by monitoring hemodynamic changes before and after interventions such as deep brain stimulation (DBS) [29], SCS [30], and rTMS [31].

Brain activity comprises a complex combination of neuronal activity, hemodynamic/metabolic regulation, and neurotransmitter release, yielding intricate multimodal information. Consequently, there is an urgent demand for a complementary neuroimaging technique to comprehensively quantify intrinsic neurophysiological and metabolic changes of brain functional activity, thereby improving diagnostic accuracy while elucidating the underlying neural mechanisms of DoC. Both EEG and fNIRS are noninvasive, portable, cost‐effective, and can be used for longitudinal monitoring. Crucially, the integration of EEG (neuronal activity) and fNIRS (hemodynamic/ metabolic activity) provides a more comprehensive assessment of residual brain function with appropriate spatiotemporal resolution than either modality alone, enhancing sensitivity to covert consciousness [32, 33, 34]. Studies demonstrate that dual modality fused features significantly enhance classification and decoding accuracy for MI and motor execution tasks [35, 36]. Notably, simultaneous photoelectric multimodal integration offers explicit physiological significance by investigation of neurovascular coupling (NVC). A recent EEG–fNIRS investigation of unresponsive intensive care unit (ICU) patients with acute brain injury demonstrated that the coupling strength between frontal neuronal oscillations (1–12 Hz) and concomitant hemodynamic fluctuations (0.07–0.13 Hz) furnishes a quantitative index for both consciousness detection and prognostic stratification [37]. Furthermore, the intrinsic portability, noninvasiveness, and resilience to motion artifacts of combined EEG‐fNIRS techniques make them particularly well‐suited for real‐time bedside monitoring with excellent ecological validity [38]. Conventional task‐based paradigms face inherent limitations, as patients may struggle to hear or comprehend instructions, or lack the cognitive capacity to complete tasks despite retaining some level of consciousness [39]. Resting‐state paradigms, which measure spontaneous brain activity in the absence of goal‐directed tasks or sensory stimuli, have been increasingly used to characterize the changes in functional connectivity (FC) in several physiological disorders. By eliminating the external intervention, resting‐state paradigms simplify the experimental design, enhance cross‐site reproducibility, and facilitate the construction of longitudinal large‐scale databases. Consequently, they are particularly suited for populations with limited task‐compliance abilities, such as young children or subjects with cognitive or motor impairments. Resting‐state studies offer valuable insights for investigating brain FC and understanding the underlying mechanisms of DoC [40]. Although resting‐state measurements provide an indirect assessment of consciousness, robust evidence from fMRI and EEG studies demonstrates their ability to accurately differentiate consciousness levels (e.g., UWS vs. MCS) [9, 41, 42] and predict long‐term recovery in severe brain injury with high precision [43]. Resting‐state paradigms eliminate the need for active patient participation, demonstrating broad applicability for assessing metabolic profile [19] and neuroelectric dynamics in DoC populations.

Although simultaneous EEG‐fNIRS integration with brain–computer interfaces (BCI) paradigms has demonstrated significant applications in stroke rehabilitation through high‐accuracy neural decoding, studies on residual consciousness of patients with DoC based on EEG‐fNIRS are limited so far [33]. In view of this, the primary objectives of this study were as follows: (1) to identify NVC biomarkers by multimodal fusion analysis; (2) to explore the key brain regions where multimodal features exhibit the strongest association with residual consciousness, and to evaluate their diagnostic efficacy in distinguishing MCS from UWS; (3) to elucidate the underlying physiological mechanisms of DoC during resting‐state conditions.

## 2 Results

### 2.1 Spectral Signatures of Neuronal Activity across Different Groups

**FIGURE 1:**
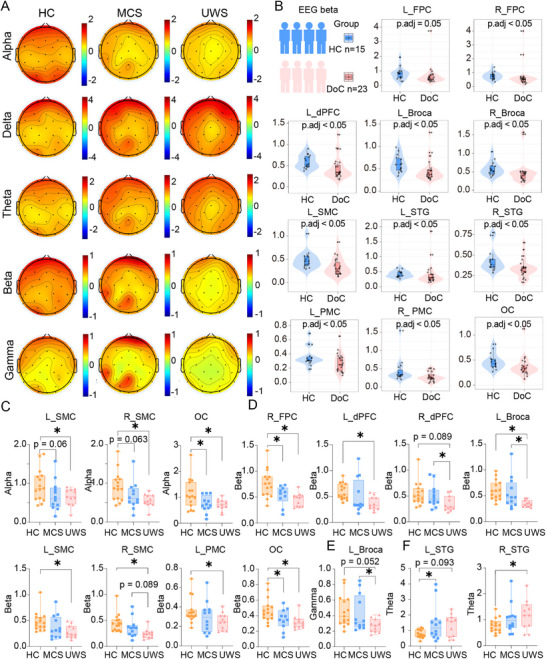
The topology map of absolute power and statistical analysis of EEG across HC, MCS, and UWS groups in frequency bands. (A) The topographical map of the power spectrum in alpha, beta, gamma, delta, and theta bands for HC, MCS, and UWS. Compared to HC, both MCS and UWS groups exhibited reduced alpha, beta, and gamma power across the whole‐brain functional area. (B) Beta‐band power between HC and DoC(MCS and UWS)across 13 ROIs. (C–F) Statistical analyses revealed frequency‐specific group differences. (C) Alpha band: OC distinguished HC from MCS, while SMC and OC discriminated HC from UWS (*p* < 0.05). (D) Beta band: R_FPC and OC showed robust HC and MCS discrimination, while R_FPC, L_dPFC, L_Broca's area, SMC, L_PMC, and OC separated HC from UWS (*p* < 0.05). Notably, R_dPFC and L_Broca's area discriminated MCS from UWS (*p* < 0.05), linking beta activity to residual attentional control in MCS. (E) Gamma band: Only L_Broca's area distinguished MCS from UWS (*p* < 0.05), with nonsignificant HC and UWS trends in R_dPFC and R_Broca's area. (F) Theta band: L_STG separated HC from MCS, while R_STG discriminated HC from UWS (*p* < 0.05). *𝑝 < 0.05. DoC, disorders of consciousness; EEG, electroencephalography; fNIRS, functional near‐infrared spectroscopy; HC, healthy controls; L_Broca's area, left Broca's area; L_dPFC, left dorsolateral prefrontal cortex; L_FPC, left frontopolar cortex; L_PMC, left premotor cortex; L_SMC, left supplementary motor cortex; L_STG, left superior temporal gyrus; MCS, minimally conscious state; OC, occipital cortex; R_Broca, right Broca's area; R_dPFC, right dorsolateral prefrontal cortex; R_FPC, right frontopolar cortex; R_PMC, right premotor cortex; R_SMC, right supplementary motor cortex; R_STG, right superior temporal gyrus; UWS, unresponsive wakefulness syndrome.

To further delineate the spectral signatures of consciousness, the EEG power spectral density (PSD) across HC, MCS, and UWS groups was compared quantitatively. Visual inspection of the group‐averaged PSD revealed that the HC cohort exhibited markedly elevated beta‐band power relative to both MCS and UWS groups (Figure 1A). To statistically validate this observation, we pooled MCS and UWS into a single DoC group and performed between‐group comparisons against HC. Beta‐band power differed significantly in 11 of 13 predefined cortical regions of interest (ROIs) (two‐sample *t*‐tests, false discovery rate (FDR)‐corrected *p* < 0.05; Figure 1B). In contrast, delta, theta, alpha, and gamma bands did not consistently separate HC from DoCs, exhibiting fewer than three ROIs with significant differences in each band. These results indicate that beta‐band oscillatory activity serves as the most robust discriminator between conscious and unconscious states, in line with prior reports linking beta rhythms to active cognitive processing and arousal mechanisms [9, 44]. Building on this finding, we next conducted a high‐resolution, ROI‐wise assessment of each frequency band's diagnostic accuracy (e.g., receiver operating characteristic [ROC] analyses) to pinpoint the cortical loci where spectral features most reliably distinguish HC from DoCs.

In the alpha band, the occipital cortex (OC) demonstrated significant group differences between HC and MCS, while SMC (left supplementary motor cortex (L_SMC), *p* = 0.060; right supplementary motor cortex (R_SMC), *p* = 0.063; and OC, *p* = 0.021) distinguished HC from UWS (Figure 1C). No brain regions reliably discriminated MCS from UWS in this frequency band. These findings align with prior research showing alpha rhythm suppression in severe DoCs, particularly in SMC and OC associated with sensory integration [44, 45]. The beta band exhibited the most robust group distinctions (Figure 1D). Right frontopolar cortex (R_FPC) and OC differentiated HC from MCS, while R_FPC, left dorsolateral prefrontal cortex (L_dPFC), left Broca's area (L_Broca's area), L_SMC, R_SMC, left premotor cortex (L_PMC), and OC discriminated HC from UWS (*p* < 0.05). Notably, right dorsolateral prefrontal cortex (R_dPFC) and L_Broca's area distinguished MCS from UWS, suggesting beta activity in frontoparietal networks (R_FPC, L_dPFC, L_Broca's area, L_PMC) may reflect residual cognitive processing in MCS. This is consistent with studies linking beta‐band synchronization to attentional control and consciousness recovery [8, 46, 47].

This supports the dominance of higher‐order association cortices (e.g., DMN), frontoparietal control network (FCN) in consciousness‐related EEG signatures, whereas PMC regions retain relatively preserved activity across unconscious states. In the gamma band, only L_Broca's area significantly separated MCS from UWS (*p *= 0.088) (Figure 1E). HC and UWS showed nonsignificant trends in L_Broca's area, possibly reflecting reduced synaptic plasticity in deep coma states. In the theta band, distinct group differences were observed primarily in prefrontal and cingulate regions (Figure 1F). Left superior temporal gyrus (L_STG) exhibited significant power spectral differentiation between HC and MCS, with HC showing higher theta power. This aligns with evidence that theta activity in L_STG correlates with working memory and attentional control, which are partially preserved in MCS but disrupted in deeper unconscious states. Notably, the right superior temporal gyrus (R_STG) demonstrated robust discrimination between HC and UWS, with UWS showing marked theta power elevation. While HC and UWS showed nonsignificant trends in other theta‐associated regions, the specific involvement of STG highlights the theta band's utility in distinguishing conscious states from severe DoC. These results contrast with alpha and beta bands, where frontoparietal networks showed broader group discrimination, suggesting theta activity may reflect domain‐specific deficits in cognitive–motor integration rather than global consciousness impairment.

### 2.2 Altered Hemodynamic Connectivity in DoC

**FIGURE 2:**
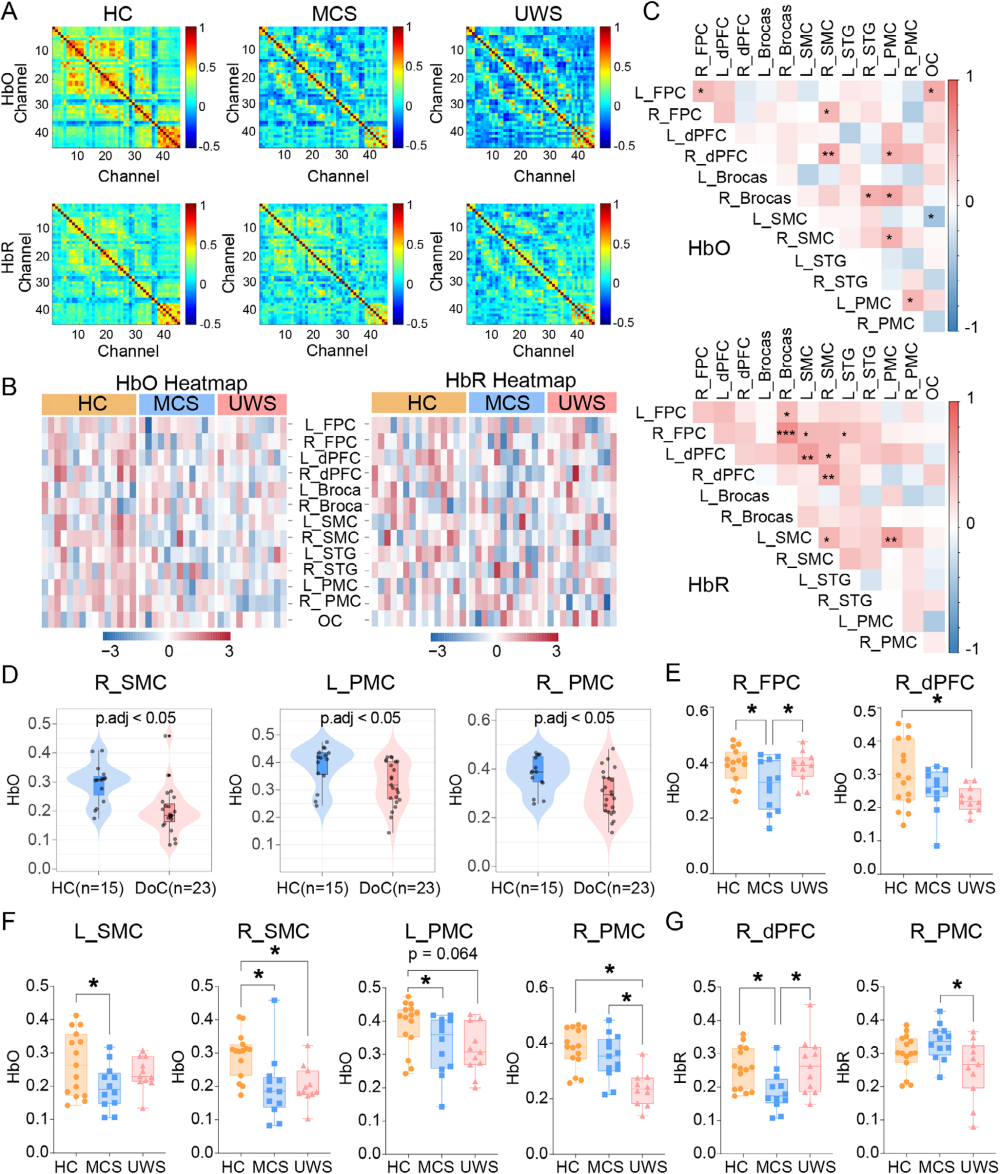
Spatial patterns of the FC in HC, MCS, and UWS groups. (A) FC maps for 45 channels of HbO and HbR, visualizing pairwise correlations across channels in HC, MCS, and UWS. Color intensity represents *z*‐scored correlation values normalized to baseline, with red denoting stronger positive correlations and blue indicating weaker or negative correlations. (B) Heatmap analyses at the individual subject depicting HbO and HbR values across 13 ROIs. Each column represents an individual participant, with a row corresponding to the ROI. Color intensity reflects *z*‐scored hemoglobin concentrations (red = higher values, blue = lower values). (C) Interregional correlation analyses within the 13 ROIs parcellation for the combined DoC group, focusing on HbO and HbR. Heatmaps display Pearson's correlation coefficients (*r*) for pairwise brain‐region interactions, with significant correlations defined as *p* < 0.05 (two‐tailed, FDR‐corrected for multiple comparisons). (D) Comparative analyses between the HC and DoC groups for HbO and HbT metrics identified regions with significant differences in the DoC group (*p* < 0.05). (E–G) Three‐group comparisons among HC, MCS, and UWS for HbO and HbR were conducted with FDR correction for significance (*p* < 0.05). *p < 0.05; **p < 0.01; ***p < 0.001. DoC, disorders of consciousness; EEG, electroencephalography; fNIRS, functional near‐infrared spectroscopy; HbO, oxyhemoglobin; HbR, deoxyhemoglobin; HC, healthy controls; L_Brocas, left Broca's area; L_dPFC, left dorsolateral prefrontal cortex; L_FPC, left frontopolar cortex; L_PMC, left premotor cortex; L_SMC, left supplementary motor cortex; L_STG, left superior temporal gyrus; MCS, minimally conscious state; OC, occipital cortex; R_Brocas, right Broca's area; R_dPFC, right dorsolateral prefrontal cortex; R_FPC, right frontopolar cortex; R_PMC, right premotor cortex; R_SMC, right supplementary motor cortex; R_STG, right superior temporal gyrus; UWS, unresponsive wakefulness syndrome.

To investigate the differences in resting‐state hemodynamic characteristics across consciousness states, whole‐brain FC matrices were computed and quantitatively compared between the HC, MCS, and UWS cohorts. Figure 2A displays group‐averaged connectivity patterns for both HbO (top) and HbR (bottom), normalized to a symmetric divergent color scale (red: stronger connectivity; blue: weaker connectivity). The HC group exhibited overall higher levels of FC compared to both the MCS and UWS groups. Despite the obvious spatial similarity between the MCS and UWS groups, the MCS group showed relatively higher FC than the UWS group in certain ROIs for HbO and HbR concentrations. Specifically, the FC were quantitatively calculated and compared across the three groups. Additionally, to further investigate the individual distribution of FC across 13 ROIs, heatmap analyses of HbO and HbR for each subject within 13 ROIs were conducted. As shown in Figure 2B, HC subjects exhibited heatmaps predominantly colored in red tones, indicating consistently higher correlation coefficient values in prefrontal, motor, and parietal networks. In contrast, MCS and UWS subjects displayed predominantly blue‐toned heatmaps, reflecting reduced correlation coefficient values in these populations.

Figure 2C presents the interregional correlation analyses across the 13 ROIs, treating MCS and UWS as a combined DoC group. For HbO, significant correlations were observed between left frontopolar cortex (L_FPC) and R_FPC (*r* = 0.429, *p* = 0.045), OC (*r* = 0.502, *p* = 0.015), reflecting FC within the frontoparietal–occipital network. R_FPC correlated with R_SMC (*r* = 0.439, *p* = 0.036), while R_dPFC showed stronger correlations with R_SMC (*r* = 0.520, *p* = 0.010) and moderate correlations with L_PMC (*r* = 0.470, *p* = 0.024). Additionally, right Broca's area (R_Broca's area) correlated with R_STG (*r* = 0.483, *p* = 0.02), L_PMC (*r* = 0.520, *p* = 0.011), L_SMC with OC (*r* = 0.457, *p = 0.02*), R_SMC with L_PMC (*r* = 0.471, *p* = 0.02), and L_PMC with right premotor cortex (R_PMC) (*r* = 0.449, *p* = 0.028), indicating interconnectedness between language, motor, and visual networks. For HbR, L_FPC correlated with R_Broca's area (*r* = 0.420, *p* = 0.046), while R_FPC exhibited strong correlations with R_Broca's area (*r* = 0.683, *p* < 0.001) and moderate correlations with L_SMC (*r* = 0.435, *p* = 0.038), R_SMC (*r* = 0.429, *p* = 0.040). L_dPFC showed strong correlations with L_SMC (*r* = 0.592, *p* = 0.003) and moderate correlations with R_SMC (*r* = 0.427, *p* = 0.042), and R_dPFC demonstrated strong connectivity with R_SMC (*r* = 0.528, *p* = 0.010). Notably, L_SMC showed strong correlations with L_PMC (*r* = 0.590, *p* = 0.003) alongside moderate correlations with R_SMC (*r* = 0.445, *p* = 0.033), highlighting motor network integrity.

It is worth noting that there were intergroup differences in HbO levels between the HC group and the DoC group in several different brain regions. Statistically significant reductions in HbO were identified in the R_SMC, L_PMC, and R_PMC in the DoC group (*p* < 0.05), consistent with the critical role of these regions in maintaining conscious awareness (Figure 2D). In contrast, no significant group‐wise differences in HbR were observed between HC and DoC across the 13 ROIs (*p* > 0.05). These findings demonstrate reduced FC in frontoparietal and sensorimotor networks in DoC, particularly in frontal–subcortical loops essential for consciousness maintenance, while suggesting a limited role of HbR alterations in the neurovascular dysfunction underlying DoC [48, 49].

Quantitatively, channel‐wise statistical analyses were performed using analysis of variance (ANOVA), and the least significant difference (LSD) test was conducted for post hoc correction. The ANOVA results revealed significant group differences in HbO within R_FPC, R_dPFC, L_SMC, R_SMC, L_PMC, and R_PMC (*p* < 0.05) (Figure 2E–G). HbR differences were evident in R_dPFC and R_PMC (*p* < 0.05), aligning with altered hemodynamic responses in frontal and motor‐related networks associated with consciousness (Figure 2G). Specifically, for HbO, significant differences between HC and MCS in R_PMC (*p* < 0.001), R_SMC (*p* = 0.002), L_SMC (*p* = 0.022), and L_PMC (*p* = 0.023), while HC and UWS differed in R_dPFC (*p* = 0.019), R_SMC (*p* = 0.034), and R_PMC (*p* = 0.001). Notably, MCS and UWS exhibited distinct HbO profiles in R_FPC and R_PMC (*p < 0.05*), with UWS showing more pronounced hypoxemia. For HbR and HC, MCS differed in R_dPFC, while MCS and UWS showed differences in R_dPFC and R_PMC (*p* < 0.05), reflecting altered oxygen metabolism in frontal networks. While no single region fully discriminated all three groups, L_PMC demonstrated robust diagnostic utility, as it reliably distinguished MCS from UWS across all hemoglobin metrics.

fNIRS‐derived hemodynamic features, particularly the differential connectivity of HbO and HbR in frontoparietal and motor networks, show promise as noninvasive biomarkers for differentiating MCS from UWS.

### 2.3 Correlation Between Neuronal and Hemodynamic Activities for Different Groups

Correlation analysis was conducted to explore the relationship between the neuronal activities and the corresponding hemodynamics. Specifically, correlation analysis between the FC of HbO/HbR and band power of EEG within different frequency bands (delta, theta, alpha, beta, and gamma) was calculated. The differences in patients with MCS and UWS revealed varying patterns of NVC in different brain regions, with significant associations mainly in the frontal and cingulate networks (Figure S1).

#### 2.3.1 The Results of the Correlation Analysis

Correlation analyses were conducted between fNIRS HbO/HbR and EEG beta‐band power in MCS and UWS patients. The results revealed distinct NVC patterns across 13 anatomically defined ROIs, with significant associations primarily in frontoparietal and temporal networks.

**FIGURE 3:**
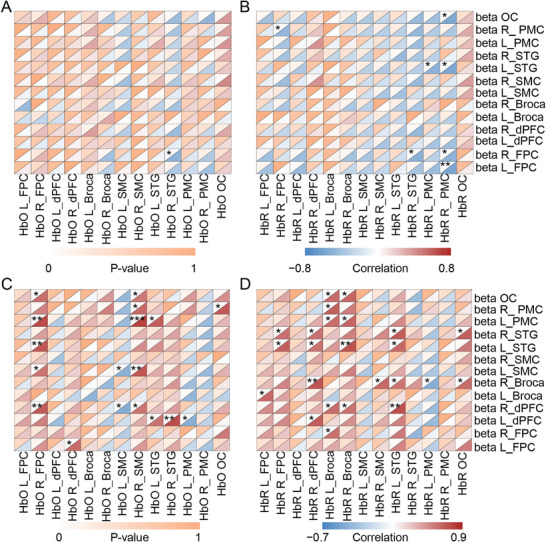
Correlation maps between fNIRS HbO/HbR and EEG beta‐band power in MCS and UWS patients across 13 anatomically defined brain regions. (A and B) present the brain region correlations and statistical analyses between HbO/HbR and beta metrics in MCS patients. (C and D) show the corresponding correlations and statistical analyses in UWS patients. Correlation coefficients (*r*) are color‐coded (red = positive, blue = negative) with size indicating magnitude; significant values (*p* < 0.05) are outlined in black. EEG, electroencephalography; fNIRS, functional near‐infrared spectroscopy; HbO, oxyhemoglobin; HbR, deoxyhemoglobin; L_Brocas, left Broca's area; L_dPFC, left dorsolateral prefrontal cortex; L_FPC, left frontopolar cortex; L_PMC, left premotor cortex; L_SMC, left supplementary motor cortex; L_STG, left superior temporal gyrus; OC, occipital cortex; R_Brocas, right Broca's area; R_dPFC, right dorsolateral prefrontal cortex; R_FPC, right frontopolar cortex; R_PMC, right premotor cortex; R_SMC, right supplementary motor cortex; R_STG, right superior temporal gyrus.

For the MCS group, FC was concentrated in the frontal and superior temporal regions. Beta_R_FPC showed significant positive correlations between HbO_R_STG (*r* = 0.61, *p* = 0.036), HbR_R_STG (*r* = 0.61, *p* = 0.033) and HbR_R_PMC (*r* = 0.60, *p* = 0.037). This aligns with studies highlighting preserved front prefrontal connectivity in MCS, linked to residual executive function. Beta power in L_STG correlated with HbR_L_PMC (*r* = 0.61, *p* = 0.033) and HbR_R_PMC (*r* = 0.69, *p* = 0.013). HbR in R_PMC is associated with beta power in L_FPC (*r* = 0.71, *p* < 0.05) and R_FPC (*r* = 0.60, *p* = 0.048), reflecting partial preservation of front‐temporal networks critical for conscious perception (Figure 3A,B).

For the UWS group, correlations were dominated by frontoparietal and premotor regions with reduced specificity. HbO_R_FPC showed robust positive correlations with beta power in R_dPFC (*r* = 0.73, *p* = 0.001), L_SMC (*r* = 0.63, *p* = 0.039), L_STG (*r* = 0.76, *p* = 0.007), L_PMC (*r* = 0.81, *p* = 0.073), and OC (*r* = 0.64, *p* = 0.033). HbO_R_dPFC showed robust positive correlations with beta power in L_FPC (*r* = 0.67, *p* = 0.025). HbO_L_SMC showed robust positive correlations with beta power in R_dPFC (*r* = 0.65, *p* = 0.029), L_SMC (*r* = 0.64, *p* = 0.042). HbO_R_SMC demonstrated strong associations with beta power in L_PMC (*r* = 0.85, p < 0.001) and L_SMC (*r* = 0.75, *p* = 0.007), consistent with global disruption of frontoparietal control networks in unconscious states. HbO_R_STG showed peak correlation with beta power in L_dPFC (*r* = 0.63, *p* = 0.038) and L_PMC (*r* = 0.73, *p* = 0.011). Beta_L_dPFC and showed peak correlation with HbO_R_STG (*r* = 0.79, *p* = 0.003) and HbO_L_PMC (*r* = 0.64, *p* = 0.035) (Figure 3C). Notably, HbR_L_FPC was positively correlated with beta power in R_dPFC (*r* = 0.67, *p* = 0.023). HbR_R_FPC demonstrated robust positive correlations with beta power in the L_STG (*r* = 0.72, *p* = 0.012) and R_STG (*r* = 0.64, *p* = 0.035). HbR_R_dPFC showed a strong positive coupling with beta power in the R_Broca's area (*r* = 0.76, *p* = 0.006). HbR_R_Broca's area was significantly correlated with beta power in the L_STG (*r* = 0.77, *p* = 0.005), the L_PMC (*r* = 0.68, *p* = 0.021), OC (*r* = 0.68, *p* = 0.020) and R dPFC (*r* = 0.63, *p* = 0.038). HbR_L_STG exhibited extensive positive connectivity, with significant correlations to beta power in the R_dPFC (*r* = 0.79, *p* = 0.004), R Broca's area (*r* = 0.62, *p* = 0.043), L_STG (*r* = 0.67, *p* = 0.023), and R_STG (*r* = 0.72, *p* = 0.012). A reciprocal connection was observed where HbR_L_PMC correlated with beta power in R_Broca's area (*r* = 0.64, *p* = 0.036). Furthermore, HbR_OC was positively correlated with beta power in the R Broca (*r* = 0.65, *p* = 0.030) and the R_STG (*r* = 0.72, *p* = 0.013) (Figure 3D).

MCS patients exhibited stronger correlations within the frontopolar‐dPFC and premotor‐temporal networks, indicating the presence of preserved functional subnetworks for minimal consciousness. In contrast, UWS patients showed broader but weaker coupling across frontoparietal and motor‐related regions, reflecting a more nonspecific neurovascular dysregulation.

#### 2.3.2 The Results of the ROC Curve Analysis

**FIGURE 4:**
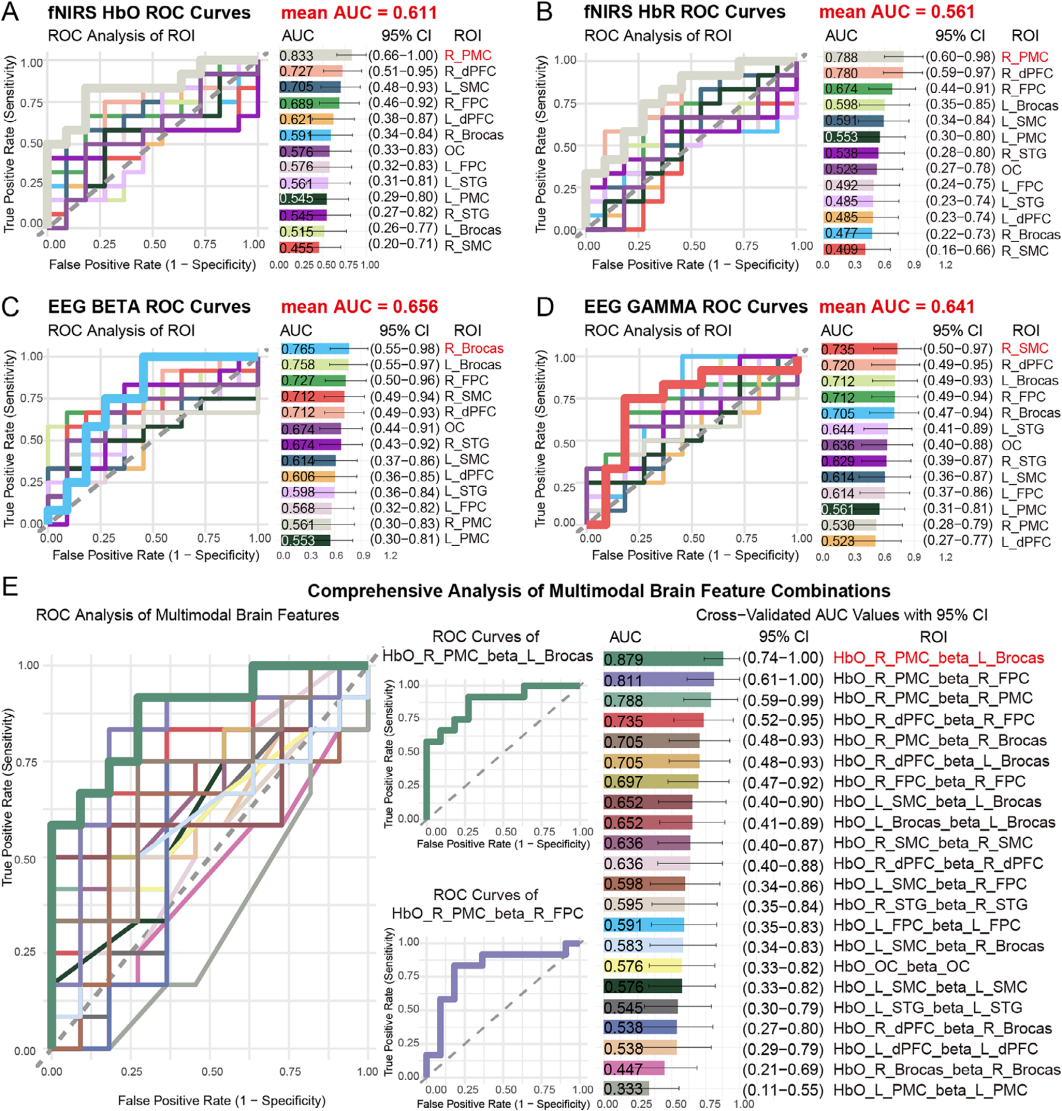
ROC curve analysis of fNIRS and EEG metrics for prognosis prediction in MCS and UWS patients. (A) ROC curves depicting the discriminative performance of HbO signals. R_PMC yielded the greatest prognostic accuracy (AUC = 0.833), followed by the R_dPFC (AUC = 0.727) and L_SMC (AUC = 0.705). (B) ROC curves for HbR metrics demonstrate that R_PMC (AUC = 0.788) and L_dPFC (AUC = 0.780) provide the most robust prognostic information. (C) ROC analyses of EEG beta‐band (13–30 Hz) power reveal significant predictive value in R_FPC (AUC = 0.727), R_dPFC (AUC = 0.712), L_Broca's area (AUC = 0.758), R_Broca's area (AUC = 0.765), and R_SMC (AUC = 0.712). (D) EEG gamma‐band (30–40 Hz) power similarly predicts outcomes, with R_FPC (AUC = 0.712), R_dPFC (AUC = 0.720), L_Broca's area (AUC = 0.712), RL_Broca's area (AUC = 0.705), and R_SMC (AUC = 0.735) all demonstrating significant discriminative ability. (E) Multimodal ROC curves for combined EEG‐fNIRS indices the optimal multimodal biomarker—HbO at R_PMC together with beta‐band power at L_Broca's area, achieving an AUC of 0.879. DoC, disorders of consciousness; EEG, electroencephalography; fNIRS, functional near‐infrared spectroscopy; HbO, oxyhemoglobin; HbR, deoxyhemoglobin; HC, healthy controls; L_Brocas, left Broca's area; L_dPFC, left dorsolateral prefrontal cortex; L_FPC, left frontopolar cortex; L_PMC, left premotor cortex; L_SMC, left supplementary motor cortex; L_STG, left superior temporal gyrus; MCS, minimally conscious state; OC, occipital cortex; R_Brocas, right Broca's area; R_dPFC, right dorsolateral prefrontal cortex; R_FPC, right frontopolar cortex; R_PMC, right premotor cortex; R_SMC, right supplementary motor cortex; R_STG, right superior temporal gyrus; ROC, receiver operating characteristic; ROI, region of interest; UWS, unresponsive wakefulness syndrome.

To explore the potential of EEG‐fNIRS characteristics for the prognosis of DoC in clinical practice, ROC curve analysis was conducted. Specifically, in this study, the ROC curve analyses for fNIRS HbO and HbR indices across 13 ROIs revealed distinct predictive utilities. For HbO, R_PMC exhibited the highest diagnostic accuracy, with an area under the curve (AUC) of 0.833, followed by R_dPFC (AUC = 0.727) and L_SMC (AUC = 0.705), indicating their significant value in prognosis prediction (Figure 4A). For HbR, R_PMC (AUC = 0.788) and R_dPFC (AUC = 0.780) emerged as key predictors, demonstrating that oxygen metabolism dysregulation in specific brain regions correlates with consciousness levels (Figure 4B).

EEG analyses highlighted the prognostic utility of beta (13–30 Hz) and gamma (30–40 Hz) frequency bands (Figure 4C,D). In the beta band, R_ Broca's area (AUC = 0.765), L_ Broca's area (AUC = 0.758), R_FPC (AUC = 0.727), R_SMC (AUC = 0.712), and R_dPFC (AUC = 0.712) showed better discrimination, suggesting that beta activity is associated with cognitive processing and arousal regulation. Similarly, gamma band analyses identified R_SMC (AUC = 0.735), R_dPFC (AUC = 0.720), L_Broca's area (AUC = 0.712), R_FPC (AUC = 0.712), R_Broca's area (AUC = 0.705), as critical, reflecting gamma oscillations’ role in neural binding and conscious perception.

Through logistic regression analysis, we found that the multimodal fusion of fNIRS HbO and EEG beta‐band indicators significantly enhanced diagnostic accuracy for DoC. To further explore the optimal diagnostic biomarkers, we combined the three brain regions with the highest AUC values from both fNIRS and EEG. The results revealed that the combination of HbO from R_PMC and EEG beta from L_Broca's area exhibited an outstanding AUC value of 0.879 (Figure 4E). This suggests that the integration of R_PMC and L_Broca's area could serve as a key diagnostic target for multimodal integration, highlighting the critical role of frontal lobe networks in consciousness assessment. In conclusion, our study identifies specific fNIRS and EEG ROIs, along with their multimodal combinations, as reliable biomarkers for differentiating MCS and UWS diagnosis.

## 3 Discussion

### 3.1 NVC Between the Neuronal Activity and the Corresponding Hemodynamics

In this study, the synchronous EEG‐fNIRS technique was used to characterize the changes of neuronal activity and the responding hemodynamics, as well as to explore the NVC relationship in patients with DoC using a resting‐state paradigm. By removing the experimental intervention, the resting‐state paradigm is valuable for exploring the intrinsic changes in brain functional activation. The current findings provide valuable insights into the field of DoC. First, resting‐state fNIRS revealed critical hemodynamic disruptions in DoC. DoCs exhibited significantly reduced FC in the L_PMC, R_dPFC, and L_SMC/R_SMC compared to HC. This aligns with the frontoparietal network's role in sustaining consciousness, where the hemodynamics‐based hypoconnectivity correlates with impaired awareness [30, 50]. Within the DoC cohort, patients with UWS exhibited lower FC than those with MCS. This aligns with prior studies demonstrating that disruptions in the DMN and FCN correlate with consciousness impairment [51, 52]. Notably, R_PMC emerged as a critical discriminator between MCS and UWS across HbO/HbR, consistent with its role in motor planning and cognitive–motor integration functions partially preserved in MCS [53, 54, 55]. These findings confirm that fNIRS studies link DMN disruption to the severity of DoC, particularly HbO depletion in the relevant cortex.

Second, beta‐Band (13–30 Hz) power emerged as the strongest electrical indicator of conscious state. HC showed significantly higher beta power in 11 of 13 ROIs compared to DoC, and frontopolar and Broca's areas further discriminated MCS from UWS. This aligns with evidence linking beta oscillations to attentional control and residual cognition in MCS [56]. In UWS, we observed marked alpha‐band suppression over sensorimotor and occipital cortices, suggesting impaired sensory integration [57]. Additionally, MCS patients retained stronger EEG‐fNIRS coupling between frontal regions and the superior temporal gyrus, indicating preserved subnetworks for conscious perception. UWS patients displayed diffuse but attenuated coupling across frontoparietal and motor regions, consistent with nonspecific neurovascular dysregulation.

The Temporal Theory of Consciousness (TTC) posits that the brain's temporal dynamics and spatial topology reflect its underlying neural activity and network properties [58]. While external stimuli can evoke brain activity, approximately 95% of neural activity is intrinsically generated as spontaneous neuronal activity [59]. Temporally, conscious awareness is associated with neural responsiveness, as indexed by event‐related potentials (ERPs) such as the P300 and N100 components [60]. These temporal dynamics arise from the synchronous and coordinated electrical activity of vast neuronal populations, with neural oscillations serving as key signatures of nonlinear brain dynamics amices [61]. Crucially, consciousness relies on the synchronization and integration of information facilitated by neural oscillations. For instance, beta and gamma oscillations exhibit spatiotemporal antagonism and constitute fundamental mechanisms for working memory. Specifically, beta oscillations are implicated in inhibitory regulation of cognitive processes; intermittent beta oscillations occur both during cognitive tasks and at rest, playing a vital role in understanding neural function and mechanisms [62]. Spatially, consciousness is supported by a distributed network encompassing the frontoparietal association cortex, cingulate cortex, precuneus, and thalamus [27]. Previous resting‐state neuroimaging studies have revealed that intrinsic network interactions, most notably within the DMN, are closely related to the levels of consciousness [48, 63].

Multimodal fusion elevated diagnostic accuracy, surpassing unimodal approaches. This synergy captures spatiotemporal consciousness mechanisms: EEG tracks millisecond‐scale neural inhibition, while fNIRS localizes vascular‐metabolic deficits, validating the TTC. Integrated EEG‐fNIRS studies have provided powerful tools for quantifying NVC under both physiological and pathophysiological conditions. In unresponsive patients with acute brain injury, the coupling strength between frontal neuronal oscillations (8–30 Hz) and concomitant hemodynamic fluctuations (0.01–0.2 Hz) has been established as a quantitative biomarker for consciousness detection and prognostic stratification [64]. These findings confirm the clinical potential of EEG‐fNIRS as a biomarker in states of brain dysfunction. A recent EEG–fNIRS study conducted in unresponsive ICU patients with acute traumatic brain injury (TBI) demonstrated that the strength of coupling between frontal low‐frequency neuronal oscillations and concurrent hemodynamic fluctuations (0.01–0.1 Hz) serves as a sensitive and quantitative biomarker for both detecting residual consciousness and predicting clinical outcome [65]. This underscores the potential of combined EEG–fNIRS not only to capture complementary aspects of brain function—where EEG provides millisecond‐scale electrophysiological dynamics, and fNIRS reflects slower, metabolically driven hemodynamic changes—but also to quantify functionally relevant NVC in severely impaired populations.

### 3.2 NVC Characteristics for Diagnosis and Prognosis of DoC

Crucially, combined HbO_R_PMC and beta_ L_ Broca's area features achieved near‐perfect classification (AUC: 0.879), outperforming unimodal metrics. This synergy arises from fNIRS capturing metabolic insufficiency in PMC while EEG detects functional network failure in frontopolar regions—dual axes of DoC pathology [66, 67]. Specifically, identification of R_PMC hypo oxygenation expands upon DMN dysfunction by highlighting motor‐cognitive nodes critical for residual awareness. Portable EEG–fNIRS systems permit real‐time, noninvasive monitoring, potentially reducing reliance on behavioral scales like the CRS‐R. fNIRS complements EEG by capturing hemodynamics in the deeper cerebral cortex, which are otherwise electrophysiological silent. For instance, diminished HbO in R_PMC likely reflects disrupted metabolic support for motor planning, whereas reduced beta power in Broca's area may index language‐network compromise in UWS.

### 3.3 The Underlying Mechanisms of DoC

DoC reflects a global shutdown of cortical activity and network integrity. Severe injury produces a broad withdrawal of excitatory synaptic input across the cortex, hyperpolarizing neurons and generating very slow rhythms. Recovery requires reengagement of the thalamocortical loops, particularly the anterior forebrain “mesocircuit” (central thalamus plus frontal cortex), which must be reactivated [3]. Critically, restoration of awareness correlates with reactivation of higher‐order fronto‐parietal networks—the DMN (anchored in medial prefrontal and posterior cingulate cortices) and the executive control network (dorsolateral prefrontal and parietal cortices). Patients emerging from DoC show gradual increases in metabolic activity and FC within these networks compared to the UWS [49].

These network disruptions produce characteristic oscillatory signatures. Beta‐band oscillations normally signal maintenance of the current sensorimotor or cognitive state. When beta activity becomes abnormally elevated or synchronized, it effectively “locks in” the status quo and impairs flexible updating. In DoC, such pathological beta persistence may help enforce the brain's fixed unresponsive state. Consistent with this, functional imaging shows that the normal anticorrelation between the DMN and task‐positive (frontal/parietal) networks—which underlies internal/external switching—is progressively lost as consciousness diminishes [68].

Motor circuits vividly illustrate these principles. The PMC and its ventral thalamic inputs carry motor commands, while a higher‐order sensorimotor circuit (including supplementary motor area, anterior cingulate, supramarginal, and temporal regions) supports conscious integration [69, 70]. In DoC, activity and connectivity in these higher‐order sensory/motor regions are disrupted, even though basic evoked responses in primary cortex may remain [71]. At the same time, NVC is severely impaired. Normally, increases in neural firing drive local hyperemia—fNIRS detects the attendant changes in oxyhemoglobin and deoxyhemoglobin as an index of cerebral blood flow. But with the cortex in a hypoexcitable state, these hemodynamic responses are blunted or delayed. Thus, an EEG recorded over a frontal region (e.g., Broca's area) may show flattened rhythms or excessive low‐frequency power, while fNIRS over PMC reveals attenuated oxygenation changes. This decoupling of electrical and blood‐flow signals highlights the core mechanism of DoC as a breakdown of normal.

### 3.4 Potential of Using Simultaneous EEG–fNIRS as a BCI for DoC

CMD represents a critical condition in which patients with severe TBI exhibit volitional brain responses to motor commands despite showing no overt behavioral signs of awareness [72]. This dissociation, observed in approximately 10%–20% of patients with DoC, carries significant prognostic and therapeutic implications, as its detection is associated with improved functional recovery and long‐term outcomes [73]. However, accurately identifying CMD remains challenging using unimodal neuroimaging approaches. In this context, the combined use of EEG and fNIRS offers a promising multimodal framework to overcome these limitations [74]. By simultaneously capturing electrophysiological dynamics and hemodynamic responses, EEG–fNIRS integration provides complementary spatiotemporal information that may enhance the detection of covert consciousness, support differential diagnosis between UWS and MCS, and ultimately facilitate the development of BCI tools for communication in behaviorally nonresponsive patients. This multimodal approach not only deepens our understanding of mechanisms but also paves the way for clinically feasible, bedside‐compatible systems capable of empowering patients with residual cognition to interact with their environment.

EEG‐fNIRS BCI has considerable potential for improving performance by measuring two different types of brain activity. Seminal work by Fazli et al. pioneered integrated EEG‐fNIRS BCI systems for MI and motor execution, demonstrating a statistically significant signal. Subsequent research refined this approach through optimized multimodal feature selection, eliminating redundant features in sensory cortical signals. Their discriminative fusion framework further enhanced MI performance by 5%, establishing neurovascular‐feature synergy as a key BCI enhancement mechanism [75].

The multimodal EEG‐fNIRS biomarkers identified in this study extend beyond diagnostic applications for DoC and offer novel avenues for the development and refinement of BCIs. First, the highly discriminative features derived from R_PMC oxyhemoglobin fluctuations and beta band power in the FPC can be directly integrated into the feature‐extraction stage of hybrid BCI systems, thereby enhancing signal‐to‐noise ratio and classification accuracy [76]. Second, by fusing electrophysiological and hemodynamic signals, our approach provides a richer, multidimensional representation of brain states in real time, which promises to improve the robustness of traditional single‐modality (EEG‐based or fNIRS‐based) BCIs under complex operational conditions [77]. Finally, the demonstrated sensitivity of beta oscillations in Broca's area among UWS patients establishes a theoretical foundation for “motor‐impaired” BCI communication channels tailored to individuals with severe motor or language deficits [78].

### 3.5 Limitations of this Study

Despite the valuable insights provided by this study, several limitations should be acknowledged. First, given the nature of DoC patients as a special population, the number of participants in this experiment was relatively small. This limited sample size may restrict the generalizability of our findings. A larger sample is often required to enhance the statistical power and reliability of results in studies involving complex patient groups [79]. Second, DoC is a post‐injury condition with complex etiologies. One of the limitations of the current study is the diverse etiologies (TBI, stroke, anoxia, etc.) of the patients with DoC, which may introduce variability [80]. Additionally, in the present study, the resting‐state data were collected only once, and the CRS‐R scores were based on the patients' conditions at that time. Fourth, although we implemented strict clinical monitoring to ensure participant stability during the recording session, the inherent fluctuations in consciousness levels that characterize DoC remain a pertinent consideration. Our study, based on a single 20‐min resting‐state acquisition, may not capture the full spectrum of diurnal variations or spontaneous state transitions that could influence biomarker reliability [81]. Although behavioral assessments conducted immediately before and after scanning confirmed that participants were in a stable state, future studies would benefit from longitudinal designs incorporating repeated measurements at different times of day and across multiple days. In addition, large‐sample, multicenter studies are needed to validate the reliability of the present methodology and to mitigate the limitations associated with small sample sizes and potential bias. Such efforts would be invaluable for establishing the temporal stability of the proposed EEG‐fNIRS biomarkers, clarifying their relationship with the dynamics of consciousness, and ultimately validating their utility for continuous bedside monitoring of patients who exhibit fluctuations in consciousness.

Although there are certain limitations, the current study should be a meaningful step in the investigation of the consciousness of patients with DoC in terms of neuronal activity, hemodynamic response, and as NVC. In further explorative studies, advanced experimental paradigms and improved processing methods should be used to delve deeper into the underlying mechanisms. More importantly, despite its imperfections, this study has significant implications for the diagnosis, prognosis, and treatment efficacy evaluation of DoC patients [82, 83]. Understanding the neural correlates of consciousness through neuro‐electrophysiological and neuroimaging techniques can serve as key biomarkers to assess recovery potential in DoC patients, thereby guiding clinical decision‐making and personalized treatment strategies. This study provides a solid foundation for future efforts in this field. Future work will include multicenter, randomized, controlled studies with long‐term follow‐up, paired with multimodal neuroimaging and biomarker analyses to clarify the mechanistic, diagnostic, and prognostic roles of EEG and fNIRS.

## 4 Conclusions

By leveraging the complementary strengths of the simultaneous EEG‐fNIRS technique, our results demonstrated that the frontoparietal and premotor network metrics offer robust, complementary biomarkers for differentiating MCS from UWS. Integrating hemodynamic and electrophysiological characteristics yielded a diagnostic accuracy (AUC = 0.879), underscoring the necessity of multimodal assessment. The current findings pave the way for portable bedside monitoring tools that can augment clinical scales, improve diagnostic precision, and ultimately guide individualized neurorehabilitation strategies.

## 5 Materials and Methods

### 5.1 Participants

In this study, 45 participants were recruited. Inclusion criteria: (1) etiology of TBI, stroke, or anoxia, and so forth, with a duration of more than 28 days and in a stable condition [84]; (2) diagnosis as UWS and MCS according to CRS‐R [85, 86]. UWS: auditory ≤ 2, visual ≤ 1, motor ≤ 2, oromotor/verbral ≤ 2, communication = 0, arousal ≤ 2. MCS: auditory = 3–4, visual = 2–5, moto*r* = 3–5, oromotor/verbral = 3, communication = 1, arousal ≤ 2. CRS‐R scoring requires that the patient's vital signs are normal and that the patient's scoring has been performed at least three times within a week before enrollment; (3) those without severe epilepsy, serious complications, and contraindications; (4) able to obtain informed consent from the legal caregivers. Exclusion criteria: (1) history of epilepsy or psychiatric or neurological disorders; (2) long‐term use of sedative or antiepileptic drugs; (3) uncontrollable infections or other serious medical diseases; (4) inability to obtain informed consent [50]. In this study, written informed consent for each subject was obtained from the patient's legal guardians. The experimental protocol of this study was approved by the IRB of Beijing Tiantan Hospital, Capital Medical University (ky2024‐043‐03). According to the quality of the acquired signal, the final study subjects included 23 DoCs (12 MCS and 11 UWS) and 15 HCs. The clinical characteristics of the patients with DoC are shown in Table 1.

**TABLE 1:** Demographic and clinical characteristics.

Characteristic	MCS (*n* = 13)	UWS (*n* = 11)	HC (*n* = 15)
Age (years), mean ± SD	44.54 ± 18.54	49.27 ± 13.44	41.93 ± 12.95
Sex (M/F), *n*	10/3	9/2	11/4
Time since injury	15 (12–25) months	10 (4–17) months	
Etiology			
TBI	6	3	
ICH	2	3	
BSH	2	3	
CI	1	1	
Anoxic	1	1	
VE	1	0	
CRS‐R score	11 [9–15]	2 [0–4]	

*Note*: The CRS‐R includes six subscales addressing auditory, visual, motor, oromotor, communication, and arousal functions, which are summed to yield a total score ranging from 0 to 23.

Abbreviations: BSH, brainstem hemorrhage; CI, Cerebral Infarction; CRS‐R, Coma Recovery Scale‐Revised; F, female; HC, healthy controls; ICH, Intracerebral Hemorrhage; M, male; MCS, minimally conscious state; TBI, traumatic brain injury; UWS, unresponsive wakefulness syndrome; VE, Viral Encephalitis.

### 5.2 Experimental Design

**FIGURE 5:**
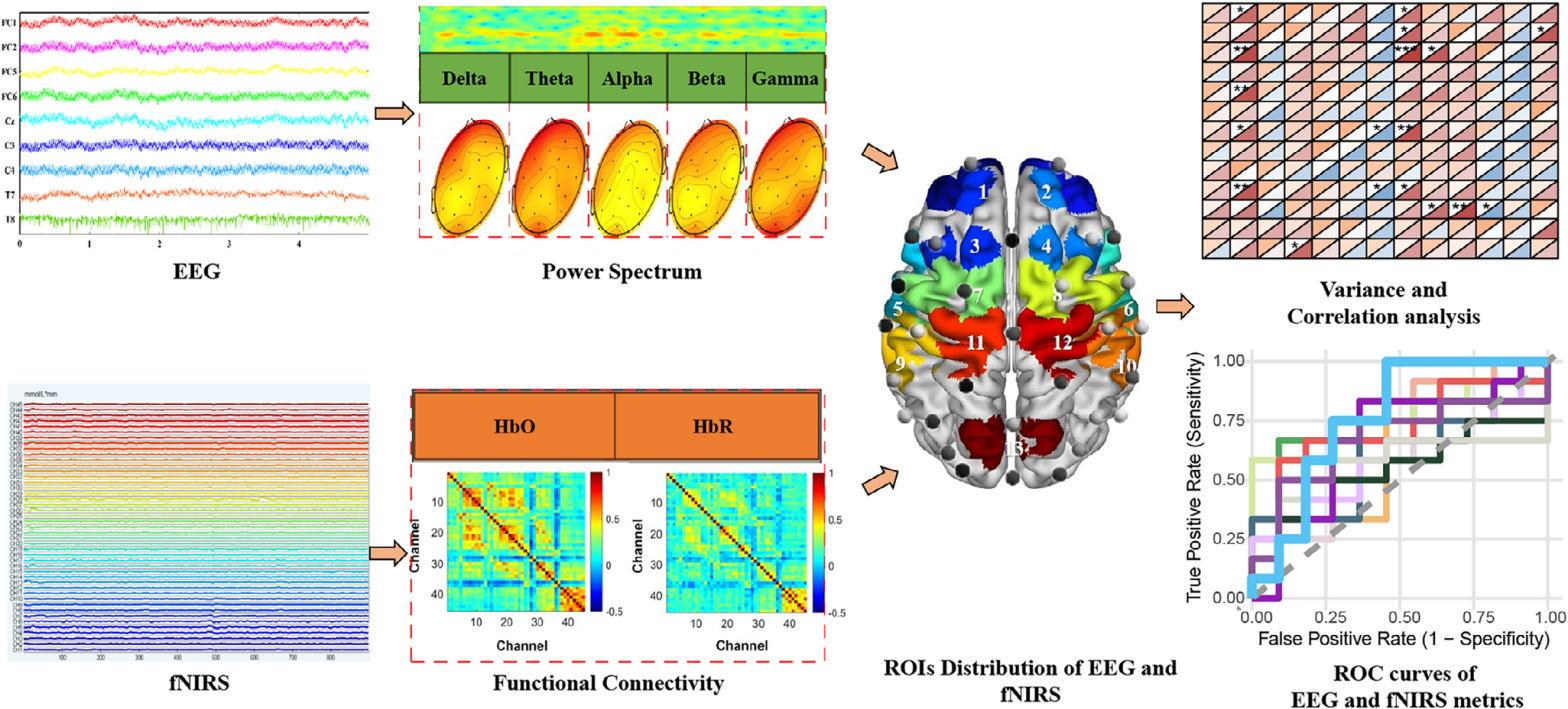
Flowchart of the EEG‐fNIRS data analysis pipeline. The simultaneously acquired raw EEG and fNIRS data from the resting‐state recording were processed concurrently. For the EEG data, the analysis focused on extracting neural oscillatory features. The data were decomposed into five canonical frequency bands: delta, theta, alpha, beta, and gamma. The PSD was calculated for each band. For the fNIRS data, the analysis focused on hemodynamic‐based connectivity. The concentration changes of HbO and HbR were used to construct FC matrices by computing Pearson correlation coefficients between the time series from all channel pairs. Subsequently, both EEG power features (for each frequency band) and fNIRS FC features (for HbO and HbR) were parcellated into 13 ROIs. The neurovascular coupling relationship was assessed by calculating correlations between the EEG spectral power features and the fNIRS‐based FC values within the same ROIs. Finally, the diagnostic utility of key features for distinguishing between patient groups was evaluated using ROC curve analysis, reporting the AUC with 95% confidence intervals. EEG, electroencephalography; fNIRS, functional near‐infrared spectroscopy; HbO, oxyhemoglobin; HbR, deoxyhemoglobin; ROC, receiver operating characteristic; ROI, region of interest.

In this study, the simultaneous EEG‐fNIRS system was used to measure resting‐state data of HCs and DoCs. Specifically, the neuronal data were obtained continuously using a wireless EEG recording system (Neuracle NeuroHUB, China) with 32 channels configured with Ag/AgCl‐pin electrodes. These electrodes were arranged in accordance with the standard international 10–20 system. An electrooculographic (EOG) electrode was placed over the outer canthus of the left eye to correct for blink artifacts. To ensure high‐quality data collection, the skin‐to‐electrode impedances were maintained below 5 KΩ, and the sampling rate of the EEG was set at 1 kHz. Correspondingly, the hemodynamic data were acquired using the NirSmartII‐3000A system (Jiangsu Danyang Huichuang Medical Equipment Co. Ltd.). Two wavelengths, 730 and 850 nm, were used to detect changes in HbO, HbR, and HbT concentrations in the brain in real time. The fNIRS system consisted of 22 sources and 15 detectors, totally generating 45 optical channels, with a source‐detector distance of 3.0 cm. The fNIRS opto‐electronic units were arranged using the 10–20 international standard EEG system. The “S” and “D” circles represent the light source and detector, respectively, and the connecting lines with numbers indicate the optical channels of fNIRS. The fNIRS system sampling frequency was 11 Hz. The 45 channels were parcellated into 13 ROIs, specifically categorized as follows: L_FPC (left frontopolar cortex, ROI 1, Channels 41, 44, and 45), R_FPC (right frontopolar cortex, ROI 2, channels 40, 42, and 43), L_dPFC (left dorsolateral prefrontal cortex, ROI 3, Channels 34, 35, and 39), R_dPFC (right dorsolateral prefrontal cortex, ROI 4, channels 31, 32, and 38), L_Broca's area (left Broca's area, ROI 5, Channels 26, 36, and 37), R_Broca's area (right Broca's area, ROI 6, Channels 15, 18, and 29), L_SMC (left primary motor cortex, ROI 7, Channels 22, 23, 25, 27, and 33), R_SMC (right primary motor cortex, ROI 8, Channels 16, 17, 19, 20, and 30), L_STG (left superior temporal gyrus, ROI 9, Channels 10, 12, 13, and 24), R_STG (right superior temporal gyrus, ROI 10, Channels 4, 5, 6, and 14), L_PMC (left premotor cortex, ROI 11, Channels 9, 11, and 21), R_PMC (right premotor cortex, ROI 12, Channels 7, 8, and 18) and OC (OC, ROI 13, Channels 1, 2, and 3) (Figure 5).

### 5.3 Experimental Paradigm

**FIGURE 6:**
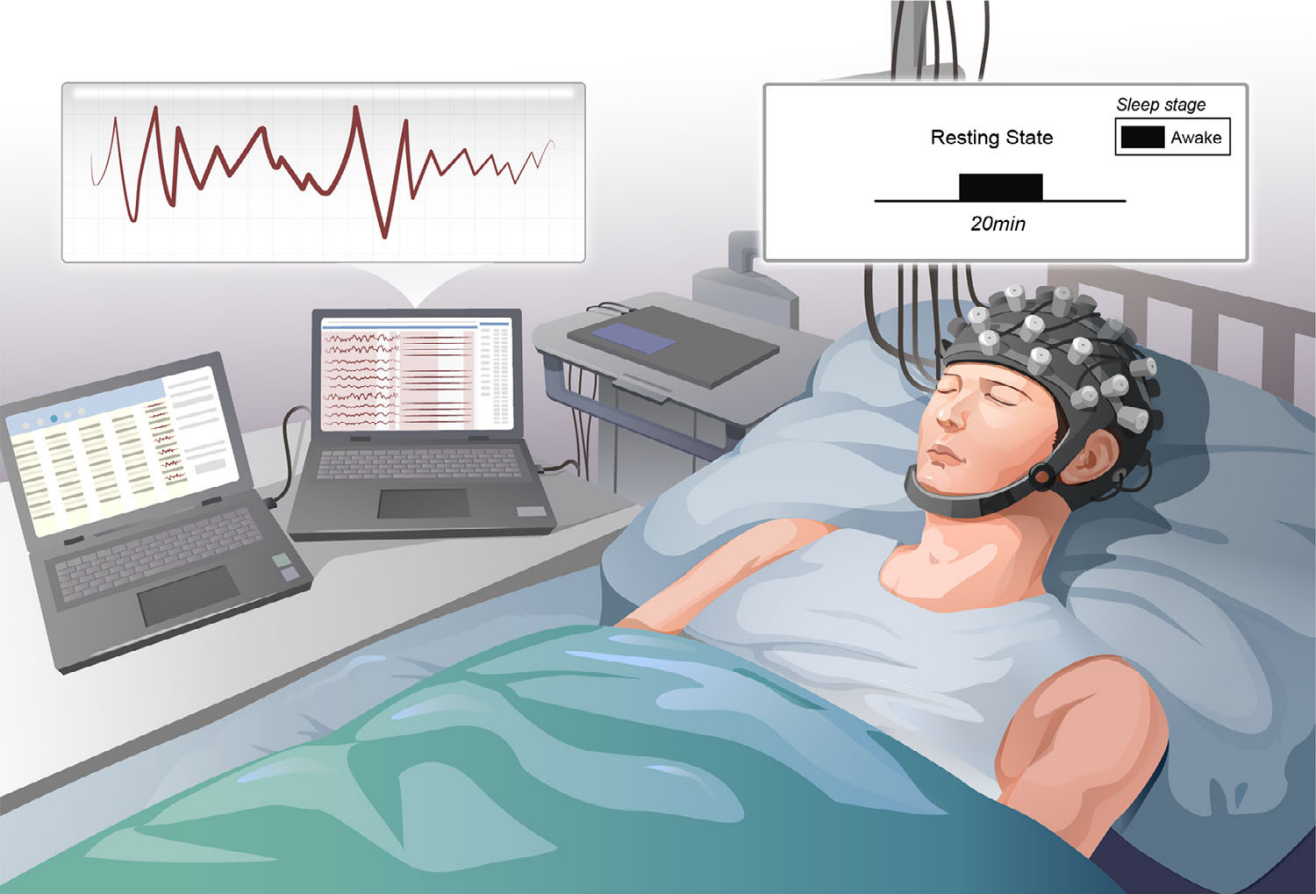
Experimental paradigm and acquisition diagram.

Participants were instructed to lie supine on the bed and remain as still as possible during the recording session, refraining from active thinking and minimizing both body and head movements. Each participant underwent a 20‐min resting‐state data acquisition in a quiet room with standard ambient lighting. The overall experimental paradigm is shown in Figure 6. To minimize the potential confounding effect of spontaneous fluctuations in consciousness levels, all data acquisitions were conducted under close clinical supervision. Immediately preceding the recording, a certified neurologist performed a brief CRS‐R assessment to confirm the patient's baseline state. Throughout the 20‐min resting‐state acquisition, the patient's behavioral stability (e.g., absence of overt arousal shifts or spontaneous motor events) was continuously monitored by the same clinician. Data collection proceeded only if the patient was deemed to be in a stable clinical state. This protocol aimed to ensure that the acquired neuroimaging data reflected a consistent and representative neural state for each participant.

### 5.4 Data Analysis

#### 5.4.1 EEG Data Analysis

The flowchart of EEG preprocessing is shown in Figure 5. The EEG data were analyzed using EEGLAB V13. At first, the raw data were band‐pass filtered between 1 and 40 Hz and band‐stop filtered between 48 and 52 (zero‐lag second‐order Butterworth digital filters). Then the data were down‐sampled to 500 Hz. After that, the EEG data were segmented into several 3‐s epochs. Furthermore, a semiautomatic procedure based on independent component analysis (ICA) was applied to identify and remove ocular artifacts and muscle artifacts [87, 88]. The scalp distribution, frequency, timing, and amplitude of EEG data were visually carefully inspected to remove bad leads, and ±100 µV extreme values were processed to remove epochs with apparent artifacts. The filtered and artifact‐corrected EEG signals were subsequently divided into five distinct rhythms depending on characteristic frequency bands of interest, including delta (1–4 Hz), theta (4–8 Hz), alpha (8–13 Hz), beta (13–30 Hz), and gamma (30–40 Hz). These brain rhythms have been extensively documented to reflect the ongoing neuronal processing in specific brain regions.

Power spectral analysis is a well‐established method for the analysis of EEG signals. The PSD reflects the ‘frequency content’ of the signal or the distribution of signal power over frequency. Set the total length of the signal to be T. Split the signal into K overlapping subsegments, each of length L, and each has M overlapping sections. For each subsegment, its power spectrum is calculated using the Discrete Fourier Transform (DFT) as shown below [89].

(1) $$PSD_{k}\;\left(f\right)=\frac{1}{L}\;{\left|F\left(f\right)\right|}^{2}$$

Where F(f) denotes the DFT of the subsegment and f denotes the frequency of the sampling point.

Then, the modified periodogram estimates from all subsegments are averaged to obtain the final PSD estimate:

(2) $$PSD\left(f\right)=\frac{1}{K}\;\sum_{k=1}^{K}PSD_{k}\left(f\right)$$

#### 5.4.2 fNIRS Data Analysis

The flowchart of fNIRS preprocessing is illustrated in Figure 1. The fNIRS data were analyzed using the FC‐NIRS package (https://www.nitrc.org/projects/fcnirs/) [90]. First, the raw acquired signals were converted to optical densities (ODs), and then to relative changes in HbO, HbR, and HbT concentrations according to the modified Beer–Lambert law [91]. After that, a band‐pass filter (0.01–0.1 Hz) was applied to remove the task‐irrelevant noise signals, such as heartbeat (0.8–1.6 Hz), respiration (0.2–0.6 Hz), and blood pressure (around 0.1 Hz) [92]. Next, motion artifacts were identified and corrected using principal component analysis (PCA). The data with large and sudden motion artifacts were rejected. Finally, 5‐min stable hemoglobin time series were extracted for further analysis.

Each optical channel was treated as a network node, and a whole‐brain FC matrix was constructed for each participant by computing Pearson correlation coefficients between the time series of all possible node pairs. This approach resulted in a 45 × 45 correlation matrix per participant. To provide an overview of FC patterns across patients with HC, MCS, and UWS, connections with Pearson correlation coefficients were visualized using the BrainNet Viewer toolbox (https://www.nitrc.org/projects/bnv/) [93]. For quantitative analysis, pairwise correlation coefficients were averaged within each of the 13 ROIs to compute regional mean connectivity. These values were then used for intergroup statistical comparisons.

#### 5.4.3 NVC Analysis

To further guarantee the synchrony of EEG–fNIRS for a reliable investigation of NVC, we extracted a continuous, artefact‐free 5‐min segment from both EEG and fNIRS datasets for subsequent analyses, during which participants exhibited minimal movement and stable consciousness levels, as confirmed by concurrent clinical assessment.

Resting‐state fNIRS analysis primarily focuses on FC metrics calculated from HbO and HbR time series. Pearson's correlation analysis was conducted to evaluate the correlations between windowed EEG spectral features (delta, theta, alpha, beta, and gamma power) and the FC values derived from fNIRS hemodynamic time series.

### 5.5 Statistical Analysis

One‐way ANOVA was used to compare the differences among the three groups (i.e., HC, MCS, UWS). To account for multiple comparisons in both the fNIRS and EEG statistical analyses, the FDR method was used to compute adjusted *p* values (*p* < 0.05). The diagnostic utility of EEG and fNIRS features in discriminating UWS from MCS was evaluated using ROC curves, implemented via the ROC package in the R statistical environment (v4.4.0) [94]. AUC values with 95% confidence intervals (CIs) were computed for all univariate feature models against binary classification outcomes (UWS vs. MCS). To evaluate the potential enhancement in discriminative performance through multimodal feature integration, we systematically compared AUC values across distinct feature sets, including EEG‐derived beta power features, fNIRS‐based HbO features, and their combinations. Furthermore, a logistic regression classifier was employed for multimodal feature integration, and its performance was rigorously evaluated using fivefold cross‐validation, with standardization parameters derived exclusively from the training set to prevent data leakage. The final reported AUC values and their corresponding 95% CIs were calculated based on aggregated predictions from all cross‐validation folds to ensure robustness and generalizability. The statistical analyses consisted of four steps: (1) comparing the absolute power in each sub‐band of the 13 ROIs; (2) comparing the mean HbO/HbR values of the defined 13 ROIs to investigate intergroup differences; (3) calculating correlation analyses of the HbO /HbR values with the delta, theta, alpha, beta, and gamma power, respectively; and 4) exploring the HC, MCS, and UWS intergroup differences.

## Author Contributions

**N**.W. conceptualized the study, drafted the manuscript, participated in the design of the figures, and conducted a literature review on EEG‐fNIRS. J.S. statistically analyzed the raw data of the article and assisted in editing and revising the manuscript. Y.H. J.S., and X.C. contributed to the editing process to enhance coherence and accuracy. D.L. J.L., T.Z., and T.C. provided feedback on clinical implications and contributed to data interpretation. Q.H., S.Z., and Y.J. conducted focused literature reviews on neuroimaging methodologies. W.M., Y.Y., and J.Z. supervised the study, offering substantial guidance on the research direction, critically reviewing the manuscript for intellectual content, and approving the final version for submission. All authors have read and approved the final manuscript.

## Funding

This work was partially supported by the Natural Science Foundation of Beijing Municipality (7232049, 7252004), CAMS Innovation Fund for Medical Sciences (CIFMS, 2019‐I2M‐5‐021), Science and Technology Innovation 2030 (2022ZD0205300), International (Hong Kong, Macao, and Taiwan) Science and Technology Cooperation Project (Z221100002722014), National Natural Science Foundation of China (82371197, 82501457) and National High Level Hospital Clinical Research Funding (2025‐PUMCH‐D‐004).

## Ethics Statement

Any aspect of the work covered in this manuscript that has involved patients with disorders of consciousness has been conducted with ethical approval approved by the ethics committee of Beijing Tiantan Hospital (KY2024‐043‐03) and the Chinese Clinical Trial Registry (ChiCTR2400085830). All procedures involving human participants complied with the ethical standards of the institutional ethics committees and the Declaration of Helsinki. Informed consent was obtained from all participants or their legal representatives before sample collection.

## Conflicts of Interest

The authors declare no conflicts of interest.

## Supporting information




**Figure S1**: Correlation maps between fNIRS HbO/HbR and EEG alpha/delta/gamma/theta‐band power in MCS and UWS patients across 13 ROIs. Correlation coefficients (r) are color‐coded (red = positive, blue = negative) with size indicating magnitude; significant values (*p < 0.0*5, Bonferroni‐corrected) are outlined in black. *p < 0.05; ** p < 0.01; *** p < 0.001. Abbreviations: L_FPC, left frontopolar cortex; R_FPC, right frontopolar cortex; L_dPFC, left dorsolateral prefrontal cortex; R_dPFC, right dorsolateral prefrontal cortex; L_Brocas, left Broca's area; R_Brocas, right Broca's area; L_SMC, left supplementary motor cortex; R_SMC, right supplementary motor cortex; L_STG, left superior temporal gyrus; R_STG, right superior temporal gyrus; L_PMC, left premotor cortex; R_PMC, right premotor cortex; OC, occipital cortex; EEG, electroencephalography; fNIRS, functional near‐infrared spectroscopy; HbO, oxyhemoglobin; HbR, deoxyhemoglobin.
**Figure S2**: ROC curves of EEG metrics for differentiating MCS and UWS across 13 brain regions. (A) EEG frequency bands of alpha: 8‐ 13 Hz. (B) EEG frequency bands of delta: 1‐ 4 Hz. (C) EEG frequency bands of theta (4‐ 8 Hz). Abbreviations: DoC, disorders of consciousness; MCS, minimally conscious state; UWS, unresponsive wakefulness syndrome; HC, healthy controls; L_FPC, left frontopolar cortex; R_FPC, right frontopolar cortex; L_dPFC, left dorsolateral prefrontal cortex; R_dPFC, right dorsolateral prefrontal cortex; L_Brocas, left Broca's area; R_Brocas, right Broca's area; L_SMC, left supplementary motor cortex; R_SMC, right supplementary motor cortex; L_STG, left superior temporal gyrus; R_STG, right superior temporal gyrus; L_PMC, left premotor cortex; R_PMC, right premotor cortex; OC, occipital cortex; EEG, electroencephalography; fNIRS, functional near‐infrared spectroscopy; HbO, oxyhemoglobin; HbR, deoxyhemoglobin; ROC, receiver operating characteristic; ROI, regions of interest.

## Data Availability

All data reported in this paper will be shared by the lead contact upon request. Any additional information required to re‐analyze the data reported in this paper is available from the lead contact upon request.
