# Supplementary material for: Cerebral Neurovascular Networks May Serve as Potential Targets for Identifying Disorders of Consciousness: A Synchronous Electroencephalography and Functional Near‐Infrared Spectroscopy Study
Source: MedComm (2020). 2025 Dec 10;6(12):e70530. doi: 10.1002/mco2.70530 (PMC12696340; doi:10.1002/mco2.70530)
Supplement: Supplementary file 1 — Figure S1: Correlation maps between fNIRS HbO/HbR and EEG alpha/delta/gamma/theta‐band power in MCS and UWS patients across 13 ROIs. Correlation coefficients (r) are color‐coded (red = positive, blue = negative) with size indicating magnitude; significant values (p < 0.05, Bonferroni‐corrected) are outlined in black. *p < 0.05; ** p < 0.01; *** p < 0.001. Abbreviations: L_FPC, left frontopolar cortex; R_FPC, right frontopolar cortex; L_dPFC, left dorsolateral prefrontal cortex; R_dPFC, right dorsolateral prefrontal cortex; L_Brocas, left Broca's area; R_Brocas, right Broca's area; L_SMC, left supplementary motor cortex; R_SMC, right supplementary motor cortex; L_STG, left superior temporal gyrus; R_STG, right superior temporal gyrus; L_PMC, left premotor cortex; R_PMC, right premotor cortex; OC, occipital cortex; EEG, electroencephalography; fNIRS, functional near‐infrared spectroscopy; HbO, oxyhemoglobin; HbR, deoxyhemoglobin. Figure S2: ROC curves of EEG metrics for differentiating MCS and UWS across 13 brain regions. (A) EEG frequency bands of alpha: 8‐ 13 Hz. (B) EEG frequency bands of delta: 1‐ 4 Hz. (C) EEG frequency bands of theta (4‐ 8 Hz). Abbreviations: DoC, disorders of consciousness; MCS, minimally conscious state; UWS, unresponsive wakefulness syndrome; HC, healthy controls; L_FPC, left frontopolar cortex; R_FPC, right frontopolar cortex; L_dPFC, left dorsolateral prefrontal cortex; R_dPFC, right dorsolateral prefrontal cortex; L_Brocas, left Broca's area; R_Brocas, right Broca's area; L_SMC, left supplementary motor cortex; R_SMC, right supplementary motor cortex; L_STG, left superior temporal gyrus; R_STG, right superior temporal gyrus; L_PMC, left premotor cortex; R_PMC, right premotor cortex; OC, occipital cortex; EEG, electroencephalography; fNIRS, functional near‐infrared spectroscopy; HbO, oxyhemoglobin; HbR, deoxyhemoglobin; ROC, receiver operating characteristic; ROI, regions of interest. [file MCO2-6-e70530-s001.docx]

**Supplemental Information**

Cerebral Neurovascular Networks May Serve as Potential Targets for Identifying Disorders of Consciousness: A Synchronous Electroencephalography and Functional Near-Infrared Spectroscopy Study

Nan Wang^1,2#^, Juanning Si^4#^, Yifang He^4^, Jiuxiang Song^5^, Xiaoke Chai^2,3^, Dongsheng Liu^7,8,9^, Jingqi Li^10^, Tan Zhang^11^, Tianqing Cao^2^, Qiheng He^2^, Sipeng Zhu^2^, Yitong Jia^2^, Wenbin Ma^1*^, Yi Yang^2,3,6*^, Jizong Zhao^2,3*^

1 Department of Neurosurgery, Peking Union Medical College Hospital, Chinese Academy of Medical Sciences and Peking Union Medical College, Beijing, China

2 Department of Neurosurgery, Beijing Tiantan Hospital, Capital Medical University, Beijing, China

3 China National Clinical Research Center for Neurological Diseases, Beijing, China

4 School of Instrumentation Science and Opto-Electronics Engineering, Beijing Information Science and Technology University, Beijing, China

5 School of Advanced Manufacturing, Nanchang University, Nanchang, Jiangxi, China

6 Brain Computer Interface Transitional Research Center, Beijing Tiantan Hospital, Capital Medical University, Beijing, China

7 Clinical College of Neurology, Neurosurgery and Neurorehabilitation, Tianjin Medical University, Tianjin, China

8 Department of Neurosurgery, Tianjin Huanhu Hospital, Tianjin, China

9 Department of Neurosurgery, Aviation General Hospital, Beijing, China

10 Hangzhou Mingzhou Brain Rehabilitation Hospital, Hangzhou, China

11 Department of Neurosurgery, The Second Affiliated Hospital of Soochow University, Suzhou, China

# These authors have contributed equally to this work.

*Correspondence

*Jizong Zhao, Department of Neurosurgery, Beijing Tiantan Hospital, Capital Medical University, No.119, South Fourth Ring Road, Fengtai District, Beijing, 100070, China. E-mail: zhaojizong@bjtth.org

*Yi Yang, Department of Neurosurgery, Beijing Tiantan Hospital, Capital Medical University, No.119, South Fourth Ring Road, Fengtai District, Beijing, 100070, China. E-mail: yangyi_81nk@163.com

*Wenbin Ma, Department of Neurosurgery, Peking Union Medical College Hospital, Chinese Academy of Medical Sciences and Peking Union Medical College, Beijing, China, Peking Union Medical College Hospital (East), No.1 Shuaifuyuan Wangfujing Dongcheng District, Beijing, 100730, China. E-mail: mawb2001@hotmail.com

## **Figure Legends:**

**
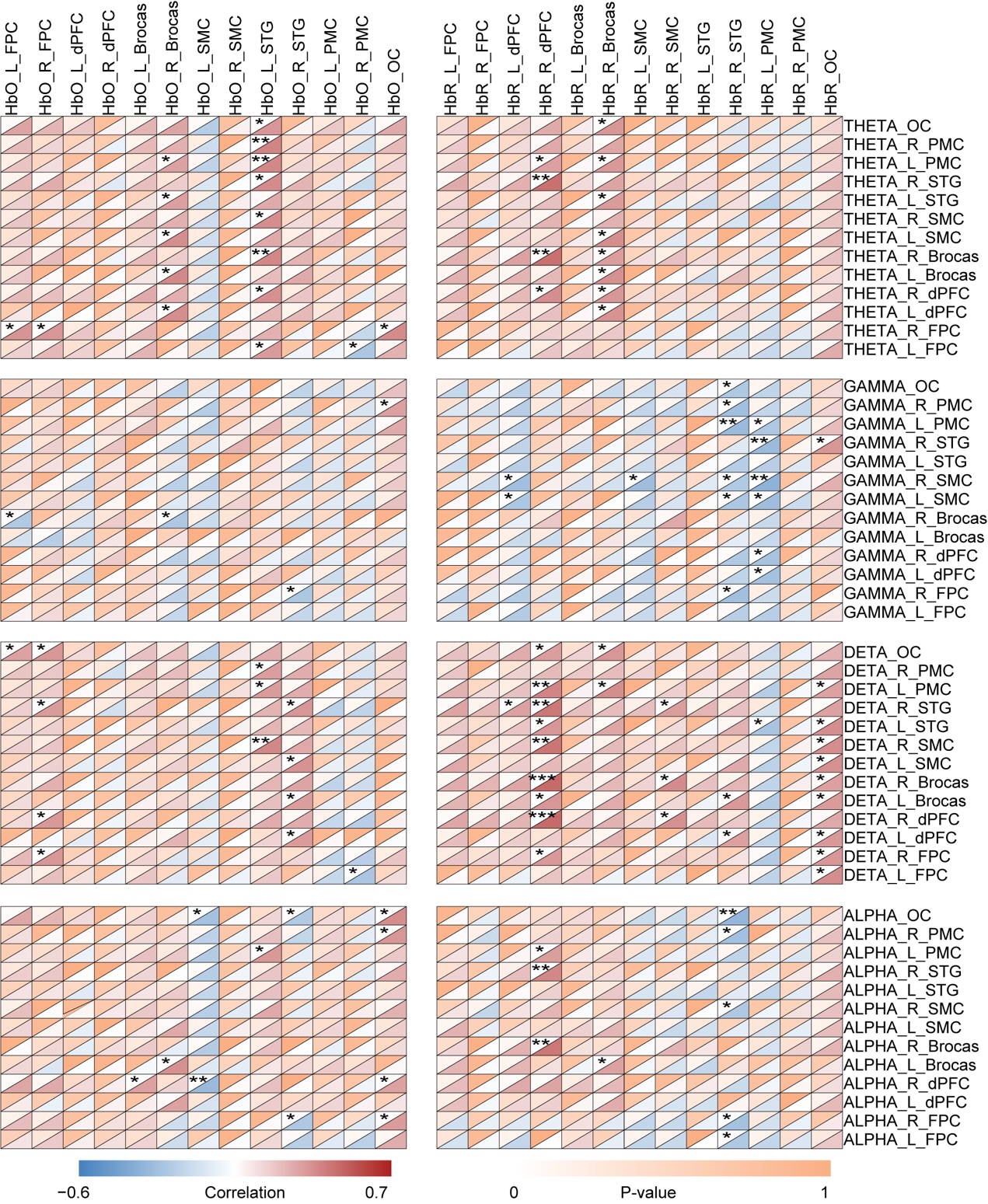
Figure S1｜Correlation maps between fNIRS HbO/HbR and EEG alpha/delta/gamma/theta-band power in MCS and UWS patients across 13 ROIs.** Correlation coefficients (r) are color-coded (red = positive, blue = negative) with size indicating magnitude; significant values (*p < 0.0*5, Bonferroni-corrected) are outlined in black. **p < 0.05; ** p < 0.01; *** p < 0.001.*

Abbreviations: L_FPC, left frontopolar cortex; R_FPC, right frontopolar cortex; L_dPFC, left dorsolateral prefrontal cortex; R_dPFC, right dorsolateral prefrontal cortex; L_Brocas, left Brocas area; R_Brocas, right Broca's area; L_SMC, left supplementary motor cortex; R_SMC, right supplementary motor cortex; L_STG, left superior temporal gyrus; R_STG, right superior temporal gyrus; L_PMC, left premotor cortex; R_PMC, right premotor cortex; OC, occipital cortex; EEG, electroencephalography; fNIRS, functional near-infrared spectroscopy; HbO, oxy-hemoglobin; HbR, deoxyhemoglobin.


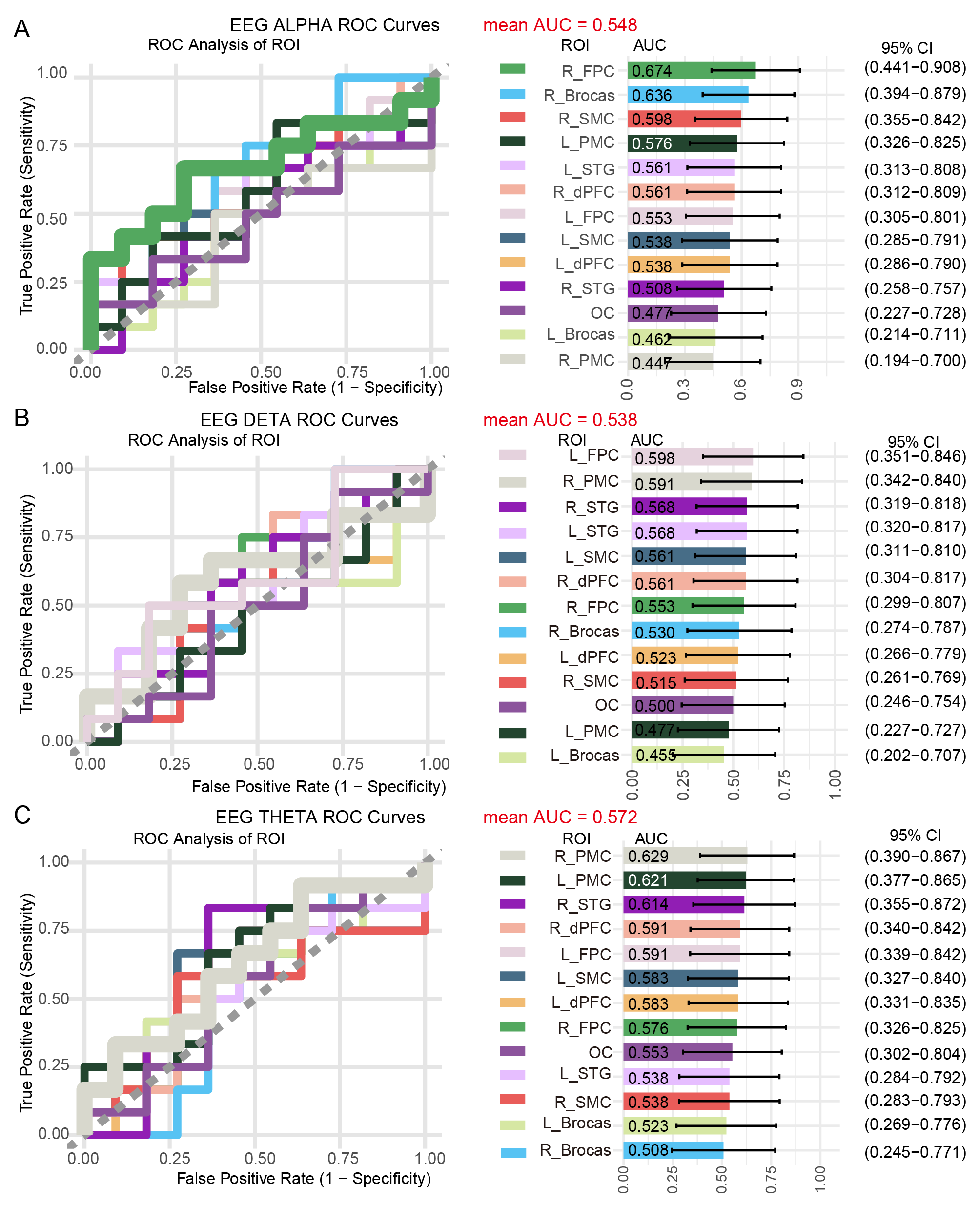
**Figure S2｜ROC curves of EEG metrics for differentiating MCS and UWS across 13 brain regions**. (A) EEG frequency bands of alpha: 8- 13Hz. (B) EEG frequency bands of delta: 1- 4Hz. (C) EEG frequency bands of theta (4- 8Hz).

Abbreviations: DoC, disorders of consciousness; MCS, minimally conscious state; UWS, unresponsive wakefulness syndrome; HC, healthy controls; L_FPC, left frontopolar cortex; R_FPC, right frontopolar cortex; L_dPFC, left dorsolateral prefrontal cortex; R_dPFC, right dorsolateral prefrontal cortex; L_Brocas, left Brocas area; R_Brocas, right Broca's area; L_SMC, left supplementary motor cortex; R_SMC, right supplementary motor cortex; L_STG, left superior temporal gyrus; R_STG, right superior temporal gyrus; L_PMC, left premotor cortex; R_PMC, right premotor cortex; OC, occipital cortex; EEG, electroencephalography; fNIRS, functional near-infrared spectroscopy; HbO, oxy-hemoglobin; HbR, deoxyhemoglobin; ROC, receiver operating characteristic; ROI, regions of interest.
